# Akita Spontaneously Type 1 Diabetic Mice Exhibit Elevated Vascular Arginase and Impaired Vascular Endothelial and Nitrergic Function

**DOI:** 10.1371/journal.pone.0072277

**Published:** 2013-08-19

**Authors:** Haroldo A. Toque, Kenia P. Nunes, Lin Yao, Zhimin Xu, Dmitry Kondrikov, Yunchao Su, R. Clinton Webb, Ruth B. Caldwell, R. William Caldwell

**Affiliations:** 1 Department of Pharmacology and Toxicology, Georgia Regents University, Augusta, Georgia, United States of America; 2 Department of Physiology, Georgia Regents University, Augusta, Georgia, United States of America; 3 Vascular Biology Center, Georgia Regents University, Augusta, Georgia, United States of America; University of Bristol, United Kingdom

## Abstract

**Background:**

Elevated arginase (Arg) activity is reported to be involved in diabetes-induced vascular endothelial dysfunction. It can reduce L-arginine availability to nitric oxide (NO) synthase (NOS) and NO production. Akita mice, a genetic non-obese type 1 diabetes model, recapitulate human diabetes. We determined the role of Arg in a time-course of diabetes-associated endothelial dysfunction in aorta and corpora cavernosa (CC) from Akita mice.

**Methods and Results:**

Endothelium-dependent relaxation, Arg and NOS activity, and protein expression levels of Arg and constitutive NOS were assessed in aortas and CC from Akita and non-diabetic wild type (WT) mice at 4, 12 and 24 wks of age. Systolic blood pressure (SBP) was assessed by tail cuff. In aorta and CC, Akita mice exhibited a progressive impairment of vascular endothelial and nitrergic function increased Arg activity and expression (Arg1 in aorta and both Arg1 and Arg2 in CC) compared with that of age-matched WT mice. Treatment of aorta and CC from Akita mice with an Arg inhibitor (BEC or ABH) reduced diabetes-induced elevation of Arg activity and restored endothelial and nitrergic function. Reduced levels of phospho-eNOS at Ser^1177^ (in aorta and CC) and nNOS expression (in CC) were observed in Akita mice at 12 and 24 wks. Akita mice also had decreased NOS activity in aorta and CC at 12 and 24 wks that was restored by BEC treatment. Further, Akita mice exhibited moderately increased SBP at 24 wks and increased sensitivity to PE-induced contractions in aorta and sympathetic nerve stimulation in CC at 12 and 24 wks.

**Conclusions:**

Over 24 wks of diabetes in Akita mice, both aortic and cavernosal tissues exhibited increased Arg activity/expression, contributing to impaired endothelial and nitrergic function and reduced NO production. Our findings demonstrate involvement of Arg activity in diabetes-induced impairment of vascular function in Akita mouse.

## Introduction

Vascular endothelial dysfunction is associated with many vascular disorders including diabetes and is accepted as a major cause of morbidity and mortality in diabetic patients. The endothelium is a key regulator of vascular smooth muscle tone through the production of nitric oxide (NO). Loss of endothelium function contributes to diabetes-induced impairment of vascular function [1]–[6]. NO, which is derived from L-arginine by NO synthase (NOS), is a critical signaling molecule regulating vascular functions.

Recent evidence indicates that elevated arginase activity contributes to impaired nitrergic and endothelium-mediated relaxation of smooth muscle in diabetes and hypertension [7], [8]. Given that NO synthase (NOS) and arginase share L-arginine as their common substrate, elevation of arginase activity can limit availability of L-arginine for NOS, thereby reducing NO production and impairing vascular function. Studies from our group and others have revealed that high glucose and diabetes induce endothelial dysfunction by increasing superoxide production and arginase activity, thereby diminishing NO levels [7], [9]–[11]. Reduction of NO production by arginase is not limited to the peripheral vascular endothelium. The corpus cavernosum (CC) of human diabetics with erectile dysfunction exhibits elevated arginase activity and diminished NO synthesis, with reduced cavernosal relaxation [12]. Additionally, inhibition of arginase has been shown to enhance NO production [1] and reduce endothelial dysfunction in hypertensive, high fat diet and diabetic states [7], [13], [14], while overexpression of arginase decreases intracellular L-arginine levels and suppresses NO synthesis [15], [16].

Two isoforms of arginase exist, arginase 1 (Arg1) and 2 (Arg2). Each is encoded by a separate gene. Both are found in vascular tissues, but their distribution is tissue- and species-dependent [17]–[19]. Elevated arginase activity/expression in vascular tissues and endothelial cells has been linked to cardiovascular diseases and inhibition of arginase restores vascular endothelial function [12], [20], [21]. Elevated Arg1 expression has been associated with cell proliferation [22] and endothelial dysfunctions during ischemia/reperfusion injury [23], aging [24], and diabetes [7]. In contrast, Arg2 appears to be involved in the pathogenesis of atherosclerosis [14], prostate cancer [25], erectile dysfunction [8], [12], [26] and diabetic renal injury [27].

To date most studies examining the mechanisms of type 1 diabetes associated vascular endothelial dysfunction have utilized streptozotocin (STZ) to induce hyperglycemia. Streptozotocin destroys pancreatic beta cells, but several disadvantages including strain dependent differential susceptibility to diabetes induction and potential extra pancreatic toxic effects, especially at high doses, have been reported [28]. Recently, mouse models that more closely resemble the natural course of the human type 1 diabetes have been developed, such as the Akita mouse. These mice have a mutation of the proinsulin 2 gene that causes protein misfolding and beta cell degeneration. They become hyperglycemic and diabetic at four-weeks of age. In this study we evaluated the role of arginase in diabetes-associated endothelial dysfunction in the Akita mice. Since up-regulation of arginase activity/expression seems to play a role in STZ diabetes-associated vascular dysfunction, we hypothesized that progression of diabetes elevates arginase activity/expression in Akita mouse, contributing to diminish NO production and impairment of vascular endothelial function in aorta and nitrergic function in the corpus cavernosum.

## Materials and Methods

### Ethics Statement

This study was carried out in strict accordance with the recommendations in the Guide for the Care and Use of Laboratory Animals of the National Institutes of Health. The protocol was approved by the Institutional Animal Care and Use Committee of the Georgia Regents University (Protocol Number: 2010-0230). All surgery was performed under a mixture of ketamine/xylazine (100∶10 mg kg^−1^, i.p.) anesthesia, and all efforts were made to minimize suffering.

### Diabetic Mouse Model

Experiments were performed in male C57BL/6-*Ins2* ^Akita^/*J* and their wild-type (WT) of the same genetic background mice (Jackson Laboratory, Bar Harbor, ME; Stock number: 003548 for Akita mouse) at four, twelve and twenty four weeks of age. Mice heterozygous for the Akita spontaneous mutation (*Ins2* ^Akita^) develop hyperglycemia around 3–4 weeks of age (description of Jackson laboratory). Mice were fasted overnight and weighed on the day of experiment. Systolic blood pressure was measured with the tail-cuff method in mice previously trained as previously described [29]. Mice were maintained on a 12-hour light-dark cycle room and were provided with food and water *ad libitum*. The Akita mice do not need supplementation of insulin because remaining levels of insulin are enough for their survival.

### Genotyping Protocol for Akita Mouse

**Figure 1:**
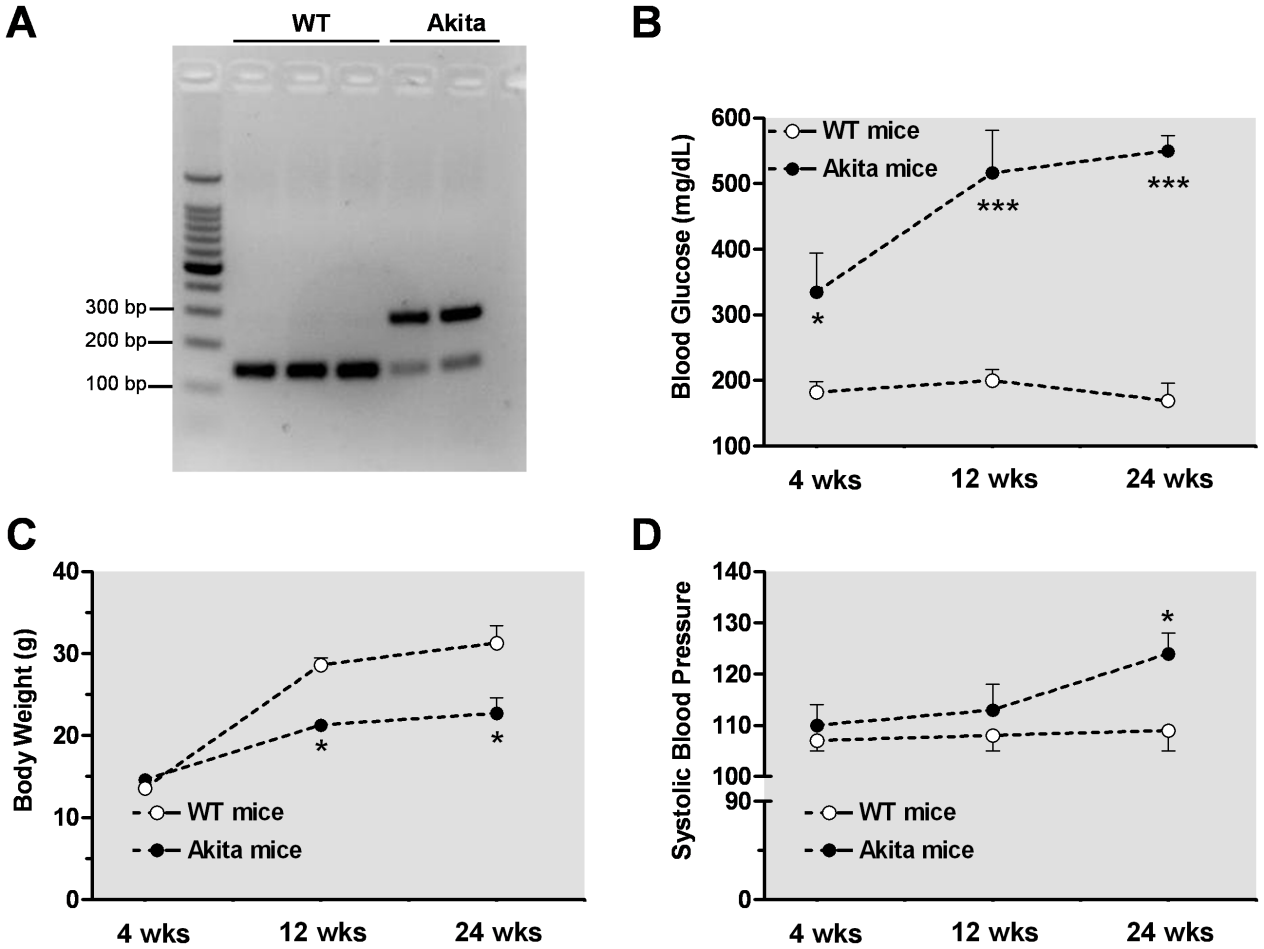
Profile of Akita mice. Genotype of mutant gene of Akita mouse by PCR analysis polymerase chain reaction (PCR) amplification was digested at 280 bp, while the control wild type (WT) gene at 140 bp (panel **A**). Differences in blood glucose levels (mg/dL, panel **B**), systolic blood pressure (SBP, panel **C**), and body weight (g, panel **D**) were observed between control WT mice and their time-course Akita diabetic mice. *p<0.05; ***p<0.001, compared with its WT group.

Genotyping was performed by polymerase chain reaction (PCR) amplification. DNA extraction from ear punch of mouse was performed following XNATG a Green Extract-N-Amp™ tissue (PCR Kit, Sigma). For genotyping, PCR analysis of the *Ins2* gene was performed (as described by Jackson Laboratory). We used restriction enzyme Fusobacterium nucleatum 4H (Fnu4H) digest and the recognition site were 5′- G CNGC - 3′ and 3′- CGNCG - 5′. Gel of 3% was used and the PCR product of the WT gene was evident at 140 bp, whereas in the mutant gene was observed at 280 bp (Figure 1A).

### Functional Studies

After the mice were deeply anesthetized, aorta and penis were rapidly excised and placed in chilled Krebs solution of the following composition (mmol/l): NaCl, 118; NaHCO_3_, 25; glucose, 5.6; KCl, 4.7; KH_2_PO_4_, 1.2; MgSO_4_ 7H_2_O, 1.17 and CaCl_2_ 2H_2_O, 2.5. After adhering fat was removed, endothelial intact aorta was cut into 2 mm rings. Connective tissue, dorsal vein and urethra were removed and mouse corpora cavernosa were cut longitudinally to obtain two identical strips (approximately 11×1×1 mm). The aortic rings or cavernosal strips were mounted under resting tension of 5.0 mN or 2.5 mN, respectively, in a myograph organ bath chambers (Danish Myo Technology A/S) filled with Krebs solution at 37°C (pH 7.4) and continuously bubbled with a mixture of 95% O_2_ and 5% CO_2_. Isometric force was recorded using a PowerLab/8SP data acquisition system (Software Chart, version 5, AD Instrument, Colorado Springs, CO, USA). The tissues were allowed to equilibrate for 1 h before starting the experiments.

After equilibration, aortic rings or cavernosal muscle strips were contracted with high KCl solution (80 mmol/l) to verify viability of preparations. After washing out KCl, cumulative concentration-response curves to acetylcholine (ACh, an endothelium-dependent vasodilator) were obtained in aortic rings or cavernosal strips after precontraction with phenylephrine (PE, α_1_-adrenergic receptor agonist). Following construction of control concentration-response curves to ACh, tissues were washed several times, incubated with an arginase inhibitor (BEC or ABH, each at 100 µmol/l for 60 min), and then a second curve was generated. Cumulative concentration-response curve to sodium nitroprusside (SNP, a NO donor) or PE were also performed in aortic rings or cavernosal tissues from WT and Akita mice.

In another set of experiments, the magnitude of relaxation induced by ACh (1 µmol/l) was monitored every 15 seconds for 5 minutes in absence or presence of an arginase inhibitor (BEC or ABH, each at 100 µmol/l) in tissues from WT and Akita mice. ABH and BEC are boronic acid analogues that specifically inhibit arginase by binding the catalytic site as the tetrahedral boronate anion, preventing the arginine hydrolysis reaction without affecting the activity of eNOS. To determine nitrergic nerve-mediated relaxation, studies were performed using electrical field stimulation (EFS) of cavernosal strips placed between two platinum ring electrodes connected to a grass S88 stimulator (Astro-Med Industrial Park, RI). EFS was conducted at 20 V, 1-ms pulse width and trains of stimuli lasting 10s at varying frequencies (1–32 Hz). To evaluate nitrergic nerve-induced relaxation, cavernosal tissues were pretreated with bretylium tosylate (30 µmol/l) and atropine (1 µmol/l) to deplete catecholamine stores and to block muscarinic receptors, respectively. Involvement of NO in EFS-induced cavernosal relaxations was confirmed using L-NAME (100 µmol/l; NOS inhibitor). To confirm the role of arginase in Akita CC, frequency-response curves were performed in the absence or presence of treatment of ABH (100 µmol/l, 60 min prior). To evaluate adrenergic nerve-mediated responses, strips were incubated with L-NAME (100 µmol/l) plus atropine (1 µmol/l) 45 min before EFS was performed.

### Arginase Activity Assay

Aortic and cavernosal tissues were collected and frozen in liquid nitrogen. Some aortic or cavernosal preparations were incubated with the arginase inhibitor BEC (100 µmol/l, for 1 hr) before they were frozen. Tissues were pulverized, homogenized in ice-cold lysis buffer (combined 1∶4 w/v with 50 mmol/l, Tris-HCl, 100 µmol/l, EDTA and EGTA, pH 7.5) containing PMSF, protease inhibitor, phosphatase inhibitors cocktail 1 and 2. Homogenates were sonicated and centrifuged at 14,000 g for 20 min at 4°C and supernatants were collected for enzyme assay. 25 µL of supernatants in triplicate were added to 25 µL of Tris-HCl 121 (50 mmol/l, pH 7.5) containing MnCl_2_ (10 mmol/l) and the mixture were activated by heating for 10 min at 55–60°C. Arginase activity was assayed by measuring urea production from L-arginine as previously described [8].

### Western Blot Analysis

Proteins (20 µg) extracted from aortas or CC tissues were separated by electrophoresis on a 10% SDS-polyacrylamide pre-cast gel and transferred to polyvinylidene difluoride (PVDF) membrane. Nonspecific binding sites were blocked with 5% of bovine serum albumin (BSA) or skim milk in Tris-buffered saline/Tween for 1 h at 24°C. Membranes were then incubated with primary antibodies (anti-arginase 1, BD Transduction Laboratories, 1∶1000; anti-arginase 2, Santa Cruz Biotechnology, Inc, 1∶250; anti-eNOS, BD Transduction Laboratories, 1∶1000; and antiphospho-eNOS at Ser^1177^ 1:1000 and anti-nNOS, 1∶4000, Cell Signaling Technology, Inc.) overnight at 4°C. After incubation with secondary antibodies, signals were visualized using an enhanced chemiluminescence kit (Amersham, Piscataway, NJ,), and quantified by densitometry. Results were normalized to β-actin protein and expressed as arbitrary unit.

### Nitric Oxide Synthase Assay

NOS enzyme activity was determined in the homogenized fractions of tissue samples by conversion of [^3^H]-L-arginine to [^3^H]-L-citrulline. Some aortic or cavernosal preparations were incubated with the arginase inhibitor BEC (100 µmol/l; for 1 hr) before they were frozen. Briefly, samples were pulverized, homogenized in buffer A (50 mmol/l Tris-HCl, pH 7.4, EDTA (0.1 mmol/l), EGTA (0.1 mmol/l) and PMSF (1 mmol/l) and then sonicated. The homogenized fraction (60 µg of protein) was incubated in buffer B containing NADPH (1 mmol/l), calmodulin (100 nmol/l), tetrahydrobiopterin (10 µmol/l), cold L-arginine (10 µmol/l), and L-[^3^H] arginine (0.6 µCi/µl) for 30 min at 37°C. The reaction was stopped after 40 min by washing with cold HEPES (50 µmol/l; pH 5.5) and EDTA (0.1 mmol/l) and L-arginine (5 µmol/l). Supernatant was added to 2-ml exchange resin columns (Dowex AG50WX-8; Dow Chemical Co. Midleand, MI), and eluted with 2 ml of washing buffer. Radioactivity corresponding to [^3^H]-citrulline content in the eluent was measured by liquid scintillation (Beckman Instruments, Fullerton, CA) as previously described.^9.^

### Statistical Analysis

Experimental values of relaxation or contraction were calculated relative to the maximal changes from the contraction produced by PE and KCl, respectively, taken as 100% in each tissue. Data were expressed as the mean ± S.E.M. Curves were fitted to all the data using nonlinear regression, and half-maximum response (pEC_50_) of each drug expressed as –log molar (M) was used to compare potency. Student’s t-test and when appropriate analysis of variance (ANOVA) followed by Bonferroni post hoc test were used to evaluate the results. A level of *p*<0.05 was considered significant. Statistical analysis was undertaken using Prim, version 3.00 (GraphPAD Software Inc., SanDiego, CA).

## Results

### Profile of Akita Mice

PCR analysis confirmed that the mutant gene of Akita mouse was observed at 280 bp, while the WT gene is seen at 140 bp (Figure **1A**). The Akita mice showed a progressive increase in blood glucose levels starting at four wks (330 mg dl^−1^) through 24 wks of age (550 mg dl^−1^) (Figure **1B**). Both Akita and WT mice had similar body weight at 4 wks of age, but WT mice gained weight more rapidly than the Akita mice as seen at 12 and 24 wks of age (Figure **1C**). Further, Akita mice had moderately increased systolic blood pressure (∼14%) at twenty four wks of age compared to WT mice (Figure **1D**).

### Increased Vascular Arginase Activity and Expression in Aorta from Akita Mice

**Figure 2:**
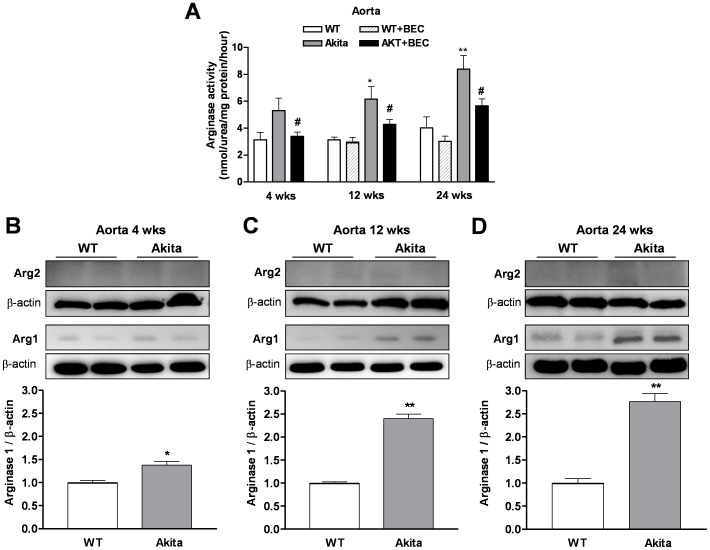
Arginase activity/expression in the aorta. Time-course analysis showed significant increases in arginase activity in diabetic mice (12 and 24 wks of age) as compared pre-diabetic mice (4 wks of age) (panel **A**). Pretreatment with (S)-(2 boronoethyl)-L-cysteine (BEC, 100 µmol/l, an arginase inhibitor) blocked this elevation. Arginase 1 (Arg1) expression in the Akita mice was slightly increased at 4 wks (panel **B**) and markedly increased at 12 (panel **C**) and 24 wks of age (panel **D**). Levels of arginase 2 (Arg2) were not different among age-matched WT and Akita mice (panel **B–D**). Values are expressed as mean ± S.E.M. of 4–5 independent experiments. * *p*<0.05; ** *p*<0.01; *** *p*<0.001compared to its WT group; ^#^ *p*<0.05, compared to Akita group.

Arginase activity was measured in aorta from age-matched WT and Akita mice. At early diabetic stage (4 wks of age), levels of aortic arginase activity tended to be increased in Akita mice (5.31±0.93 nmol/mg protein/hour) compared with those of WT mice (3.14±0.54 nmol/mg protein/hour). However, aortic tissues from Akita mice had significant elevations of arginase activity at 12 wks (6.16±0.94 nmol/mg protein/hour) and 24 wks (8.40±1.1 nmol/mg protein/hour) of age compared with those of age matched WT mice (2.95±0.37 and 4.02±0.83 nmol/mg protein/hour, for 12 and 24 wks of age, respectively) (Figure **2A**). Treatment with the arginase inhibitor BEC (100 µmol/l, for 1 hr) significantly reduced arginase activity in early diabetic (3.39±0.32 nmol/mg protein/hour) and later stage diabetic aorta from Akita mice (4.29±0.35 and 5.66±0.53 nmol/mg protein/hour at 12 and 24 wks of age, respectively). No changes were observed in basal levels of arginase activity in WT tissue at 12 and 24 wks of age treated with BEC (in the absence: 3.14±0.19 and 4.02±0.83 nmol/mg protein/hour and presence of BEC: 2.95±0.37 and 3.01±0.40 nmol/mg protein/hour, for 12 and 24 wks of age, respectively (Figure **2A**).

To determine whether increases in aortic arginase activity in Akita mice is associated with elevated protein levels of Arg1 or Arg2, we performed western blots. Our results showed a significant increase in Arg1 expression in aorta from Akita over WT mice at pre-diabetic (increased by 1.38 fold, Figure **2B**) and diabetic stage (increased by 2.40 and 2.77 fold at 12 and 24 wks of age, respectively, Figure **2C and 2D**). Arg2 was barely detectable at all time points in aorta of control WT and Akita mice and levels were not different among age matched groups (Figure **2B–D**), suggesting Arg1 as the predominant vascular isoform.

### Increased Vascular Arginase Activity and Expression in Corpus Cavernosum (CC) from Akita Mice

**Figure 3:**
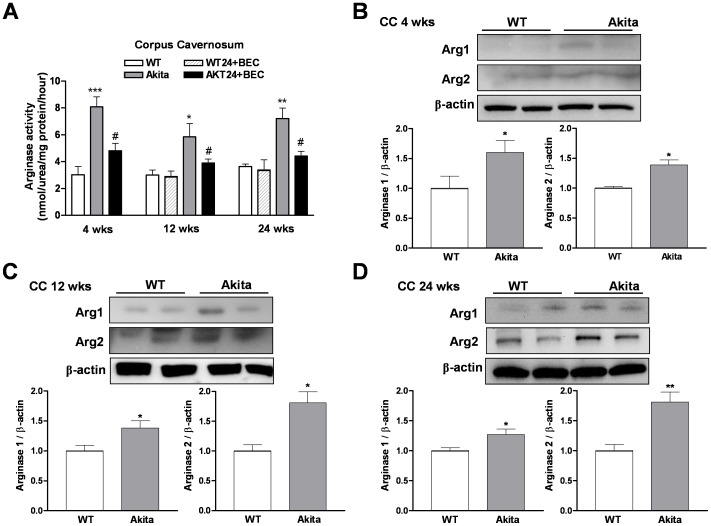
Arginase activity/expression in the corpus cavernosum (CC). Time-course analysis of cavernosal arginase activity at pre-diabetic stage (4 wks of age) and diabetic (12 and 24 wks of age) stage (panel **A**). Pretreatment with (S)-(2 boronoethyl)-L-cysteine (BEC, 100 µmol/l, an arginase inhibitor) significantly prevented these increases. Levels of both arginase 1 (Arg1) and arginase 2 (Arg2) were markedly increased in pre-diabetic (panel **B**) and diabetic (panel **C** and **D**) Akita mice. Values are expressed as mean ± S.E.M. of 4–5 independent experiments. * *p*<0.05; ** *p*<0.01; *** *p*<0.001compared to its WT group; ^#^ *p*<0.05, compared to Akita group.

Levels of arginase activity were markedly increased in cavernosal tissues from pre-diabetic and diabetic Akita mice compared with those of age matched WT mice (3.03±0.61, 3.0±0.37 and 3.64±0.18 nmol/mg protein/hour *vs* 8.08±0.75, 5.86±0.98 and 7.22±0.77 nmol/mg protein/hour for 4, 12 and 24 wks of age, respectively, Figure **3B**). Treatment of CC from Akita mice with the arginase inhibitor BEC (100 µmol/l, for 1 hr) markedly reduced arginase activity (4.82±0.54, 3.90±0.30 and 4.43±0.35 nmol/mg protein/hour for 4, 12 and 24 wks of age, respectively). No alterations were observed in basal levels of arginase activity in WT tissue at 12 and 24 wks of age treated with BEC (2.90±0.40 and 3.4±0.87 nmol/mg protein/hour, for 12 and 24 wks of age, respectively, Figure **3A**). In contrast to aorta, both arginase isoforms (1 and 2) were increased in CC tissues at 4, 12, and 24 wks of age compared with its age-matched WT mice (Figure **3B, 3C and 3D**).

Since Arg1 and Arg2 are highly expressed in liver and kidney in STZ-diabetic mice (Morris et al., 2011; Romero et al., 2008), we also examined arginase activity in these tissues from Akita mice. In early stage of diabetic Akita mice, we observed that arginase activity tended to increase in liver (by1.33-fold), but this evidence was much clearer in kidney (by 3.01-fold increase) compared with that of WT mice (Figure **S1**). In diabetic liver and kidney from Akita mice at 12 and 24 wks of age, significant increases in arginase activity were observed compared with those of age matched WT mice (increased in liver by 1.68- and 1.48-fold and in kidney by 3.12- and 2.94-fold, respectively) (Figure **S1**).

### Endothelium-Dependent Relaxation on Aorta and Corpora Cavernosa

**Figure 4:**
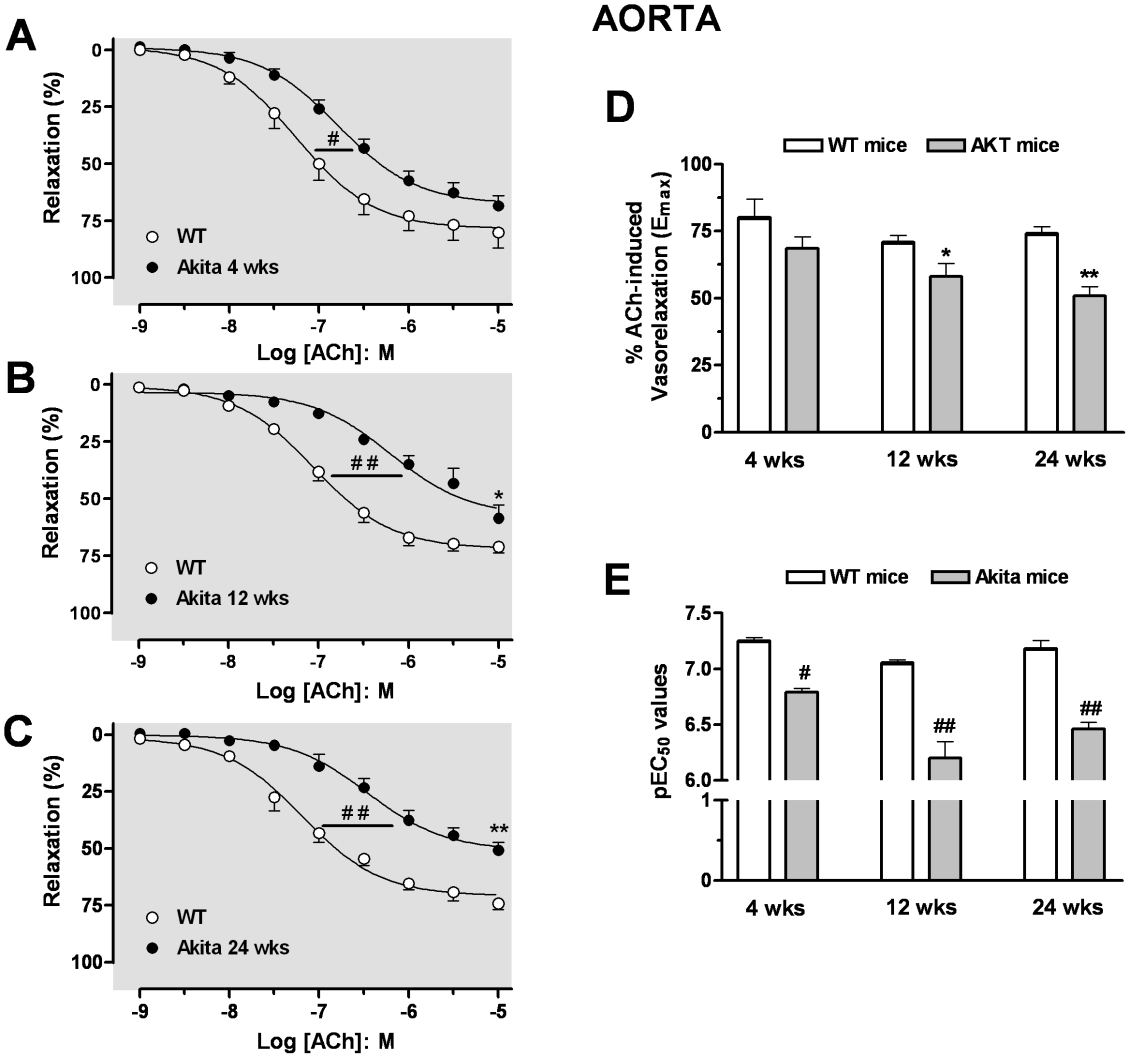
Impairment of endothelium-dependent relaxation in the aorta. Concentration-response curves to acetylcholine (ACh, 0.001–10 µmol/l) were analysed at 4 (panel **A**), 12 (panel **B**), and 24 wks of age (panel **C**) in aorta precontracted with phenylephrine (PE). The maximal efficacy (E_max_) and potency (pEC50) values of ACh-induced aortic relaxation are presented in panels **D** and **E**. Data were calculated relative to the maximal changes from the contraction produced by PE (1 µmol/l), which was taken as 100%. Data are the means ± S.E.M. of 5–7 experiments. ^#^ *p*<0.05; ^##^ *p*<0.01, indicates differences in pEC_50_ values of the dose-response curves; * *p*<0.05; ** *p*<0.01, compared with its WT mice group.

Vascular endothelial function was determined in age-matched WT and Akita mice by relaxation of aortic rings and CC strips to ACh. Cumulative addition of ACh produced relaxation of PE-contracted aorta or CC strips in age-matched WT and Akita mice. Aortas from 4 wk Akita mice exhibited less sensitivity to ACh (pEC_50_: 6.79±0.13) compared to those of WT mice (pEC_50_: 7.25±0.06), but the maximal relaxation response (E_max_) values to ACh were not different between vessels of Akita and WT mice (Figure **4A**). In aortas from Akita mice, a progressive impairment in ACh-induced relaxation pEC_50_ and E_max_ values was observed between 12 and 24 wks of age compared with those of WT mice (pEC_50_ values of 6.20±0.12; 6.46±0.06 and E_max_ values of 58±5%; 51±3% for Akita mice at 12 and 24 wks of age *vs* pEC_50_ values of 7.06±0.02; 7.18±0.07 and E_max_ values of 71±3%; 74±3% for WT mice at 12 and 24 wks of age, respectively) (Figure **4B, 4C, 4D, 4E**).

**Figure 5:**
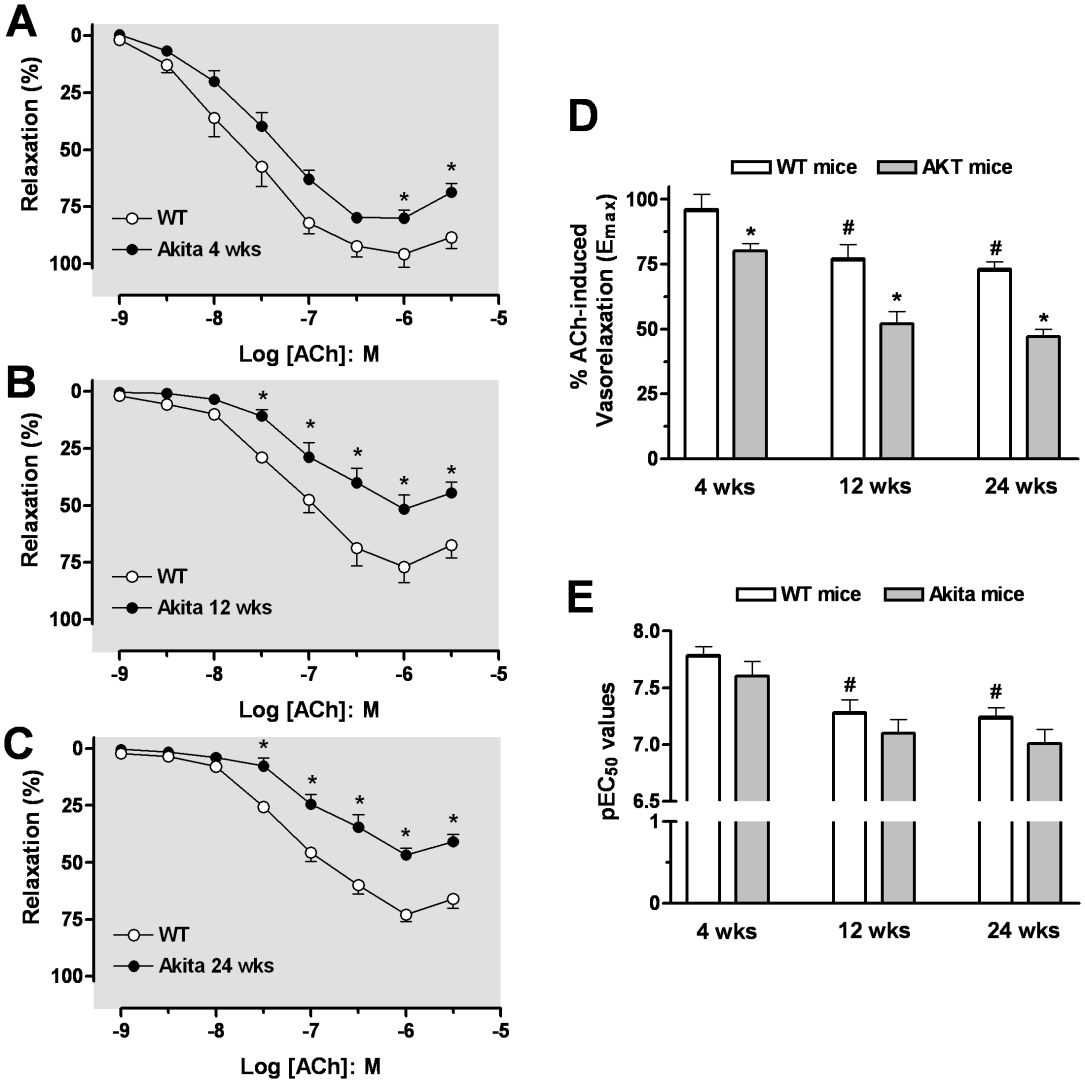
Impairment of endothelium-dependent relaxation in the CC. Concentration-response curves to acetylcholine (ACh, 0.001–3 µmol/l) were analysed at 4 (panel **A**), 12 (panel **B**), and 24 wks of age (panel **C**) in CC precontracted with phenylephrine (PE, 10 µmol/l). The maximal efficiency (E_max_) and potency (pEC_50_) values of ACh-induced aortic relaxation of WT and Akita are presented in panels **D** and **E**. Data were calculated relative to the maximal changes from the contraction produced by PE, which was taken as 100%. Data are the means ± S.E.M. of 4–7 experiments. * *p*<0.05, compared with its WT mice group.^#^ *p*<0.05, compared to WT mice at 4 wks of age.

The maximal relaxation to ACh in CC from WT or Akita mice was observed at 1 µmol/l, with a higher concentration of ACh tending to contract the CC. The sensitivity (pEC_50_) and E_max_ values of 4 wk WT CC to relaxation by ACh was significantly greater than those of CC from WT mice at 12 and 24 wks of age (Figure **5D, 5E** **)**. The CC from Akita mice had reduced E_max_ values at 4, 12 and 24 wks compared to CC of WT mice (Akita: 80±3%; 52±6%; 47±3% *vs* WT: 96±6%; 77±7%; 73±3%, respectively) (Figure **5A–D**). Atropine pretreatment (1 µmol/l) blocked ACh-induced relaxation in aorta and CC (data not shown). Thus, both aorta and CC of Akita mice showed impaired responses to muscarinic receptor stimulation. Although at 4 wks of age impaired endothelium-dependent relaxation was observed in aorta and CC from Akita mice, the impairment was more evident at 12 and 24 wks, suggesting a progressive diabetes-associated endothelial dysfunction in Akita mice.

In addition, endothelium-independent relaxations in aorta and CC tissue induced by the NO donor, sodium nitroprusside (SNP) were not different in pEC_50_ and E_max_ values between age matched WT and Akita mice at age matched 4, 12 and 24 wks of age (Figure **S2**).

### Nitrergic Relaxation Responses are Decreased in Corpus Cavernosum (CC) from Akita Mice

**Figure 6:**
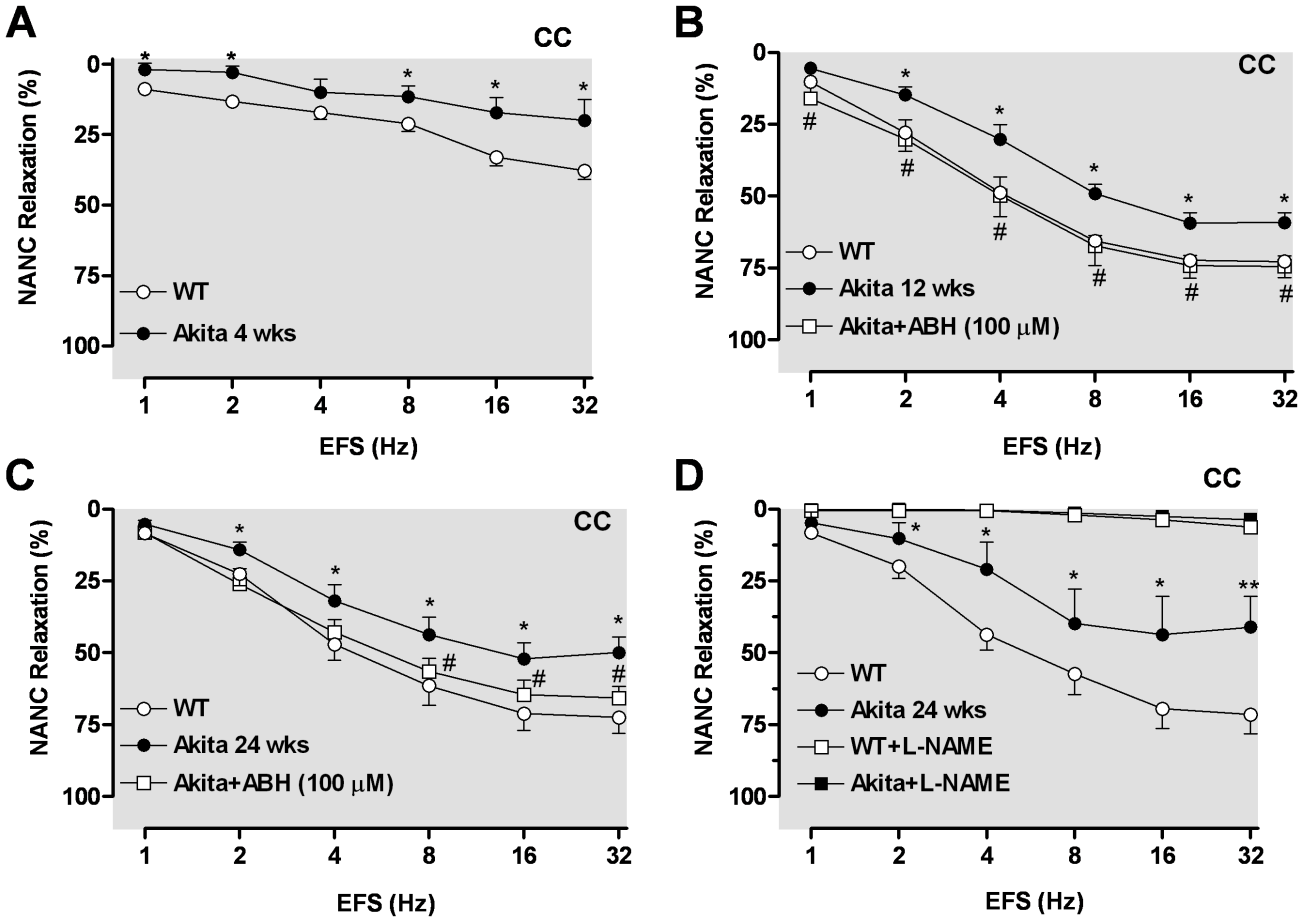
Reduced cavernosal nitrergic relaxation is enhanced by inhibition of arginase. EFS-induced nitrergic relaxations (1–32 Hz) were performed at 4 (panel **A**), 12 and 24 weeks of age. Pretreatment with an arginase inhibitor ABH (100 µmol/l) significantly attenuated impaired nitrergic relaxation in Akita mice at 12 (panel **B**) and 24 (panel **C**) wks of age, whereas treatment with L-NAME (100 100 µmol/l) fully abolished EFS-induced nitrergic relaxation in CC of WT and Akita mice (panel **D**). Data represent the means ± S.E.M. of 5–6 experiments. * *p*<0.05 and ** *p*<0.01, compared with its respective frequency in WT mice; ^#^ *p*<0.05, compared with its respective frequency in Akita mice.

**Figure 7:**
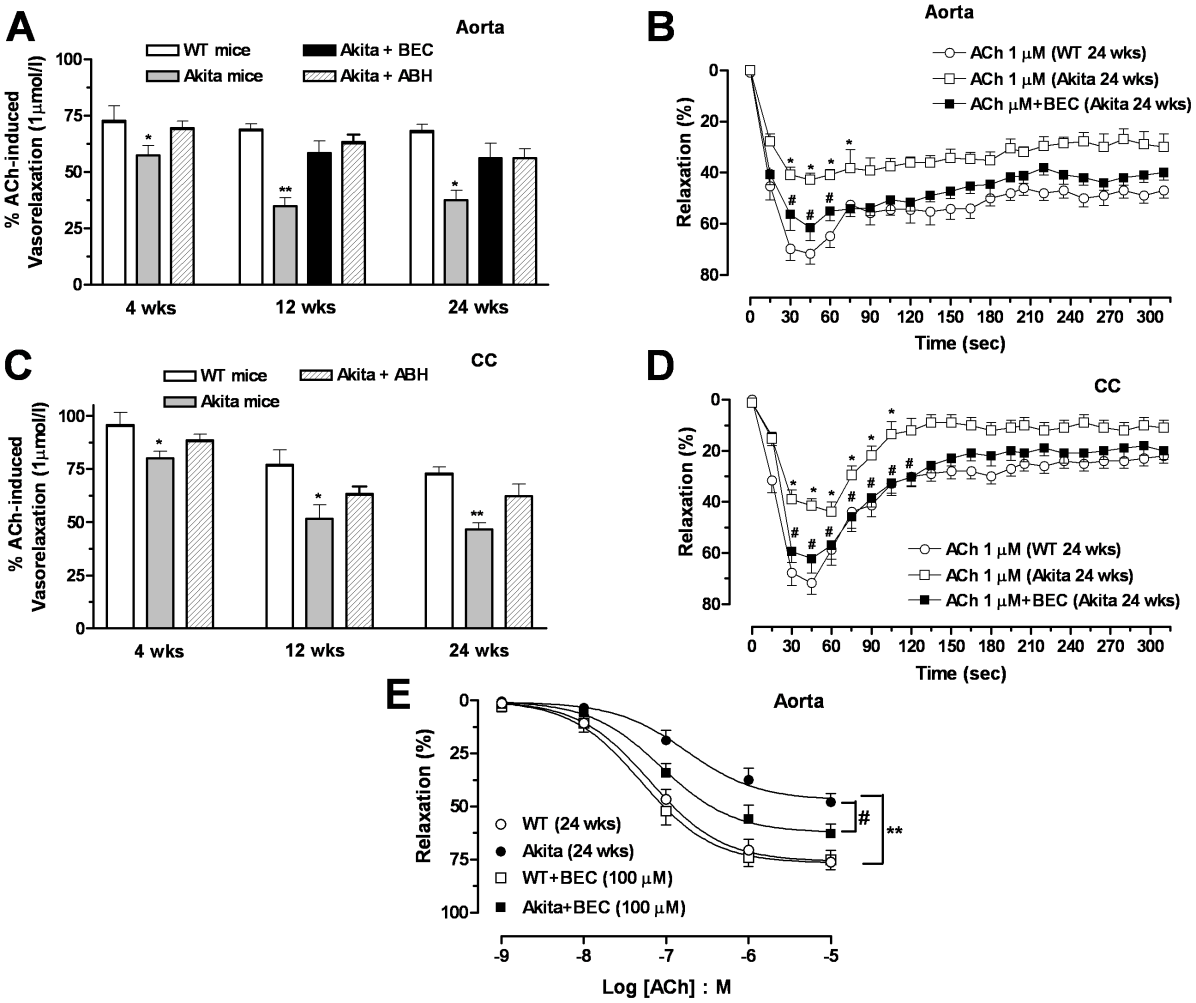
Inhibition of Arginase enhances endothelium-dependent relaxation. Relaxation was induced by a single dose of ACh (1 µmol/l) in aorta (panel **A** and **B**) and corpus cavernosum (CC, panel **C** and **D**) in the absence or presence of an arginase inhibitor, BEC or ABH (100 µmol/l each) in wild type (WT) and Akita mice. Concentration-response curve to ACh (0.001–10 µmol/l) in aorta of WT and Akita mice at 24 wks of age in the absence or presence of BEC (100 µmol/l) (panel **E**). Data represent the means ± S.E.M. of 4–6 experiments. * *p*<0.05 and ** *p*<0.01, compared with control WT mice; ^#^ *p*<0.05, compared with its respective concentration or time course in Akita mice.

To evaluate effects of nitrergic nerve stimulation, cavernosal strips from WT and Akita mice were incubated with bretylium tosylate and atropine (*see Methods*). Strips were then contracted with PE and frequency-dependent relaxation to electrical field stimulation (EFS, 1–32 Hz) was evaluated. A small EFS-induced relaxation was observed in CC at 4 wks of age in both WT and Akita mice *vs* older mice. However, the relaxation evoked by EFS in CC of 4 wk Akita mice was significantly lower than in WT CC (Figure **6A**). The CC of Akita mice at 12 and 24 wks showed a marked reduction of EFS-induced relaxations compared to age-matched WT controls (Figure **6B, 6C**). The EFS-induced relaxation in CC from 12 wk Akita mice was fully restored by ABH treatment (Figure **7B**); this treatment also significantly enhanced relaxation at 8 to 32 Hz in CC from 24 wk Akita mice. These results confirm that diabetes impairs nitrergic CC relaxation in Akita mice. Further, EFS-induced relaxation was completely abolished in the CC after incubation with L-NAME (100 µmol/l) in both groups, confirming the nitrergic nature of these responses (Figure **6D**).

### Inhibition of Arginase Enhances Endothelium-Dependent and Nitrergic Relaxation in Akita Mice

To determine the involvement of arginase in diabetes-induced endothelial and nitrergic dysfunction in Akita mice, aortas and CC were then exposed to the arginase inhibitor BEC or ABH (100 µmol/l) for 1 hr prior to ACh- or EFS-induced relaxation. In some experiments we used BEC, ABH or both arginase inhibitors to examine the role of arginase in diabetes. Since maximal relaxation of these tissues was reached at 1 µmol/l of ACh, effects of arginase inhibition were examined at this ACh concentration. Our data show that in aortas from 4 wk Akita mice, ABH fully restored endothelial function (Figure **7A**). While a significant aortic endothelial dysfunction was observed in Akita mice at 12 and 24 wks of age, inhibition of arginase attenuated or diminished this impairment.

In another set of experiments, we examined the time course of vascular relaxation in aorta from diabetic Akita mice (24 wks of age). In aortic rings from WT mice, maximal relaxation in response to ACh (1 µmol/l) was about 71%, which was reached in 30 s, waning to ∼56% by 90 s. The maximal response to ACh was smaller in Akita aorta compared with WT, reaching values of 41% at 30 s and returning to ∼36% by 90 s (Figure **7B**). Pretreatment of aortas with BEC increased the magnitude of ACh-induced relaxation in Akita aorta over the period of 15 to 75 s (Figure **7B**). Additionally, incubation of aorta from Akita mice with BEC significantly enhanced relaxation for the full ACh dose-response curve (Figure **7C**). However, exposure of WT aorta to BEC did not improve relaxation responses, indicating arginase inhibition does not affect vascular function at a basal non-diabetic state.

Similarly in cavernosal tissues, inhibition of arginase by ABH attenuated impairment of ACh-induced relaxation Akita CC (4, 12 and 24 wks of age) (Figure **7D**). Additionally, in CC strips of 24 wk Akita mice the magnitude of relaxation to ACh (1 µmol/l) was also less compared to WT mice. Incubation of Akita CC with BEC increased relaxation responses at 30 thru 120 s (Figure **7E**). The magnitude of relaxation to ACh in CC from WT mice was not altered by exposure to BEC (data not shown).

### Contractile Responses Induced by Phenylephrine (PE) on Aorta and Corpora Cavernosa

PE caused concentration-dependent contractions in aorta and CC from WT and Akita mice at 4, 12 and 24 wks. Earlier and later diabetic aortas from Akita mice exhibited increased sensitivity to PE-induced contractions compared to those of age-matched WT mice (pEC_50_ in Akita: 7.29±0.02; 7.67±0.06; 7.62±0.09 and pEC_50_ in WT: 7.00±0.05; 7.28±0.07; 7.11±0.05 for 4, 12, and 24 wks of age, respectively, Figure **S3A–C**). Additionally, increased E_max_ values to PE were observed in Akita mice compared with age-matched WT mice (12 and 24 wks) (Figure **S3B–C**). Also, increased sensitivity to PE-induced contraction was observed in CC from Akita mice compared to its age-matched WT mice (pEC_50_ in Akita: 6.41±0.03; 6.06±0.03; 6.01±0.04 and pEC_50_ in WT: 6.17±0.05; 5.84±0.02; 5.74±0.04 for 4, 12, and 24 wks, respectively) (Figure **S3D–F**).

### Contractile Responses Induced by Adrenergic Nerve Stimulation on Corpora Cavernosa

Contractile responses of CC from Akita mice to EFS (at 2–64 Hz) were significantly greater than that observed in WT tissues at 12 and 24 wks (Figure **S3G–I**). However, these responses were abolished by catecholamine depleting agent, bretylium tosylate (30 µmol/l), confirming that these contractile responses are mediated by catecholamine release due to nerve stimulation (Figure **S3I**). Since responses to CC of Akita were greater compared to WT tissue, augmentation of contractile responses to EFS in CC of Akita mice are most likely due to enhanced sympathetic nerve function, possibly differences in prejunctional events.

### Decreased Protein Levels of eNOS and nNOS in Akita Mice

**Figure 8:**
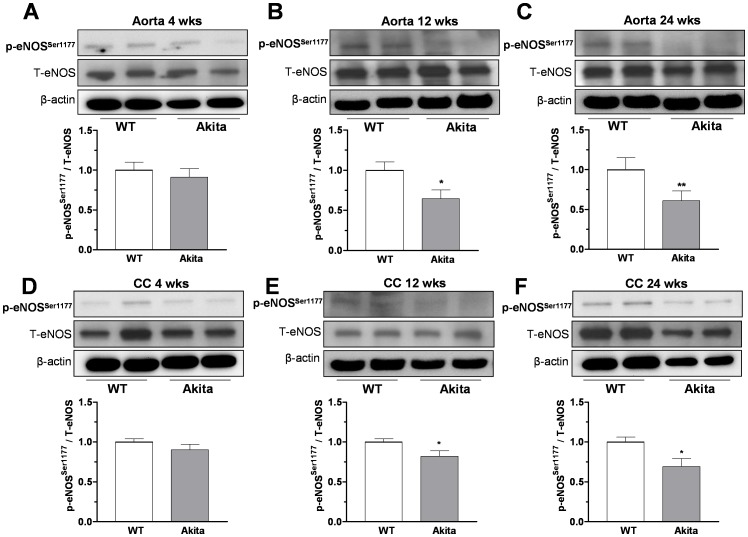
Protein levels of phospho-eNOS^Ser^ ^−1177^ in aorta and corpus cavernosum (CC). Levels of phospho eNOS (at Ser-1177), total eNOS, and β-actin were determined in aorta (panel **A–C**) and CC (panel **D–F**) from wild type (WT) littermate and Akita mice at 4, 12 and 24 weeks of age. Representative blots are shown in the top of each panel. Densitometric analysis was carried using Gene Snap software, results normalized to β-actin and expressed as arbitrary units. Data represent the mean ± S.E.M. of 6 experiments (each group). * *p*<0.05; ** *p*<0.01, compared to WT mice.

**Figure 9:**
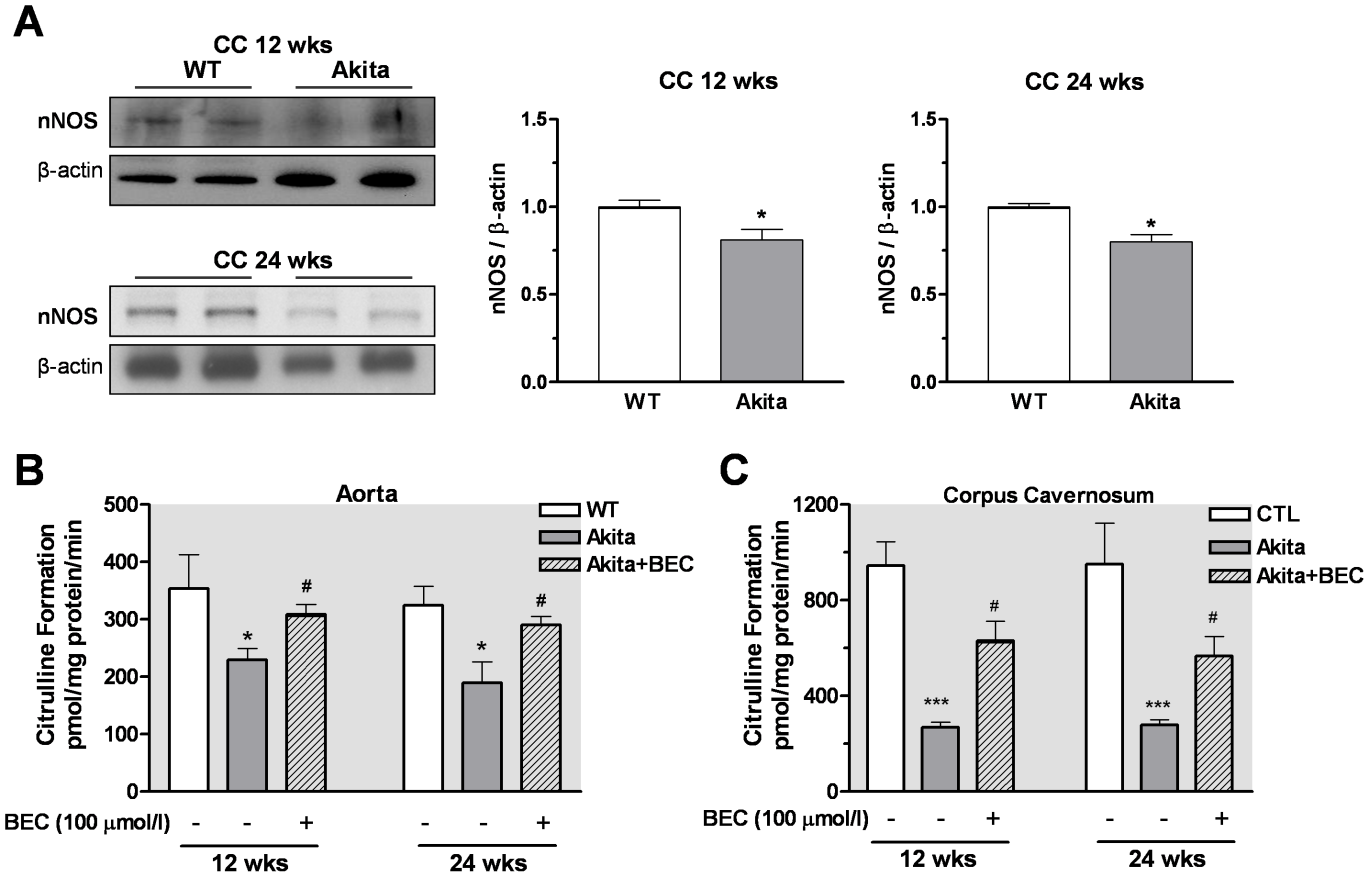
Levels of nNOS protein and NOS activity in the corpus cavernosum (CC). Protein levels of nNOS were analysed in non-diabetic and diabetic cavernosal tissues at 12 and 24 weeks of age. Representative blots of nNOS (panel **A**). Densitometric analysis was carried our using Gene Snap software, results normalized to β-actin and expressed as arbitrary units. NOS activity was determined by the conversion of _L_-arginine to _L_-citrulline in the absence and presence of the arginase inhibitor BEC (100 µmol/l) in aorta and CC from control WT and Akita mice (Panel **B**). Values are expressed as mean ± S.E.M. of 4 experiments per group. * *p*<0.05; *** *p*<0.001, compared to WT mice; ^#^ *p*<0.05, compared with its Akita control.

Diabetes has been shown to reduce levels of active phosphorylated form of eNOS (Ser-1177) in vascular tissues (26). While no significant differences in levels of phospho-eNOS at Ser-1177 were observed at 4 wks between aortic or CC tissues from Akita mice *vs* those of WT mice (Figure **8A, 8D**), reduced expression levels of p-eNOS at Ser-1177 were observed in aorta (Figure **8B, 8C**) and cavernosal tissues (Figure **8E, 8F**) of Akita mice at 12 and 24 wks compared to age matched WT mice. Because only small nitrergic relaxation responses were observed in CC at 4 wks of age and tissue mass was small, we only determined the expression of nNOS in CC at 12 and 24 wks of age. A significant reduction in nNOS expression was observed in CC from Akita mice compared to that of age matched WT mice (Figure **9A**).

### Arginase Inhibition Prevented Decrease of NOS Activity/NO Production in Akita Mice

NOS activity was measured in aorta and CC from WT and Akita mice in the absence or presence of the arginase inhibitor BEC (100 µmol/l). It was examined only at 12 and 24 wks of age because greater impairment of endothelial function is observed in aorta and CC from Akita mice at those ages. Calcium-stimulated NOS activity and conversion of _L_-arginine to _L_-citrulline was significantly lower in both aorta (Figure **9B**) and CC (Figure **9C**) from Akita mice compared to those of WT mice at 12 and 24 wks of age. Inhibition of arginase with BEC resulted in significantly enhanced conversion of _L_-arginine to _L_-citrulline (NO production) in both tissues from Akita mice. Incubation of both tissues of WT mice with BEC did not alter NOS activity (data not shown).

## Discussion

Since potential nonspecific tissue toxicity is reported in the streptozotocin model of type 1 diabetes, identification of a mouse model that mimics human diabetic cardiovascular disease remains a significant challenge. The present study characterized the vascular function of aorta and corpus cavernosum (CC) at three progressive time points of diabetes in Akita mice. Our study validates the use of Akita spontaneous type 1 diabetic mouse for gaining insight into mechanisms that contribute to the development of diabetes-induced vascular dysfunction. We examined aorta and CC tissues from Akita mice at an early (4 wks old) and later diabetic stage (12 and 24 wks old). We found a progressive elevation of activity and expression levels of arginase and impairment of vascular endothelial and nitrergic function. We observed impaired vascular endothelial and nitrergic function in aorta and CC, and increased protein expression of Arg1 in aorta and both Arg1 and Arg2 in CC of Akita compared to WT mice. We also observed reduced NO production and NOS function in both vessels of 12 and 24 wk old Akita vs WT mice. Since NO production is necessary for relaxation in aortic and penile smooth muscles, impaired production or biological activity of NO released from vascular endothelium (eNOS) or nitrergic nerves (nNOS) would result in vascular and CC dysfunction in Akita mice. Based on our use of specific inhibitors of arginase, we clearly demonstrated that arginase is a key factor in the pathogenesis of diabetes associated to endothelial dysfunction and autonomic neuropathy.

Increased arginase activity is linked with dysfunction of vascular tissue and endothelial cells in aging, hypertension, atherosclerosis, hyperglycemia, and diabetes [7], [14], [30]–[32]. Moreover, in diabetes both endothelial and nitrergic dependent relaxations in vascular tissues are impaired [33], [34]. In order to examine the role of arginase during progressive stages of diabetes, we performed studies in 4, 12 and 24 week old Akita mice. While significant impairment to ACh-induced relaxation was observed in aorta and CC from Akita mice at 12 and 24 wks compared to age matched WT controls, only a slightly reduced sensitivity of aorta to ACh and diminished maximum relaxation of CC were observed in 4 wk Akita mice. Our results also showed marked reduction of nitrergic relaxation responses in CC from Akita *vs* WT mice at 12 and 24 wks of age. These findings indicate a progressive impairment of endothelial and neuronal NO-induced relaxation in Akita vessels, which is correlated with elevated vascular arginase activity and expression in aorta and CC of Akita mice.

Arginase critically regulates NO production by competing with NOS for their common substrate, _L_-arginine. Arginase catalyzes hydrolysis of _L_-arginine to form _L_-ornithine and urea. Depending on the disease state and tissue, Arg1, Arg2 or both may be elevated and exert a prominent action. Previous studies in our lab and by others have demonstrated that both arginase isoforms are involved in vascular dysfunction in aorta, coronary artery and CC from aging and spontaneously hypertensive rats and diabetic mice [7], [8], [13], [24], [35]. We have demonstrated that streptozotocin (STZ) diabetic mice with partial knockout of Arg1 are protected against diabetes-induced vascular dysfunction compared to WT mice [36]. Additionally, knockdown of Arg1 can prevent endothelium-dependent dysfunction in aorta of aging rats [24], [37]. In contrast, Arg2 gene deletion protects against the impairment of cavernosal relaxation in STZ diabetic mice [8].

In the present study, we found that inhibition of arginase by either ABH or BEC partially restored relaxation response to ACh in aorta and CC from Akita mice at 4, 12 and 24 wks of diabetes. Further, the magnitude and duration of the responses of a single dose of ACh was markedly enhanced by the arginase inhibitor in aorta and CC of Akita mice, suggesting that arginase contributes to the diminished vasorelaxation. Since the arginase inhibitors did not enhance relaxation responses to ACh in aorta and CC from non-diabetic WT mice, it appears that arginase activity in healthy WT tissues is low enough to allow sufficient supply of _L_-arginine for normal ACh-induced NO production and vasorelaxation. Thus, the efficacy of an arginase inhibitor is only evident when it is applied to tissues in which activity of arginase is elevated enough to affect NO production. Treatment with ABH also restored the nitrergic relaxation response in CC of Akita mice compared to that of WT mice. Our findings agree with previous studies in which arginase inhibitors prevented or reversed vascular dysfunction in animal models of aging [21], [38], hypertension [13], [31], and diabetes [7], [8], [36].

The Akita mouse, characterized by chronic hypoinsulinemia and hyperglycemia, is reported to develop spontaneous diabetes by four weeks of age [39]. Our study confirmed this diabetic state in 4 wk old Akita mice, and that glucose levels progressively increase over our period of observation. Yang and Chen [40] have reported that elevated oxidation stress appears primarily responsible for vascular dysfunction in an Akita model of diabetes. Furthermore, enhanced activity of NADPH oxidase and reduced levels of NOS co-factor tetrahydrobiopterin, necessary for NOS coupling, were observed. Enhanced oxidative stress can elevate tissue arginase activity and expression [7], [9], [41], [42].

Because increased arginase activity can reduce NO production and vascular relaxation in aorta and CC, it is plausible that enhanced vascular arginase function reduces _L_-arginine availability to NOS and thereby compromises NO-mediated relaxation. In the present study, homogenized aorta and cavernosal tissue from Akita mice at 12 and 24 wks of age showed a significant decrease in NOS activity/NO production compared to control WT tissue. Additionally, reduction of _L_-citrulline/NO formation from _L_-arginine involves elevated arginase activity since inhibition of arginase partially restored NO formation in Akita vessels. Reduced NOS function in vascular tissues has been shown in STZ-diabetic models [43]–[46]. Our present findings suggest that in Akita mice increased arginase activity/expression decrease NOS function by reducing the availability of _L_-arginine, their common substrate. This is in accord with previous studies in STZ diabetic models in which inhibition of arginase increases NOS function and NO levels in vascular tissue [7], [8].

Previous studies also have shown that posttranslational modification of NOS regulates its activity [47]. Reduction in eNOS function via NOS modifications or protein-protein interactions are involved in the pathogenesis of endothelial and nitrergic dysfunction. Both eNOS and nNOS isoforms are tightly regulated and produce NO in endothelial cells and autonomic (nitrergic) nerve endings of the penis [46]. Phosphorylation of eNOS regulates its activity and NO production and involves actions of protein kinases and phosphatases at specific sites [48]. Earlier studies reported diabetes-related changes in eNOS phosphorylation in vascular tissues. Aortic and cavernosal preparations from diabetic mice and carotid plaques from diabetic patients display decreased levels of phospho-eNOS at Ser^1177^ [8], [49]–[51]. Our present study indicates that Akita mice express reduced levels of phospho-eNOS at Ser^1177^ in aortic and cavernosal tissues at 12 and 24 wks of age, but not at 4 wks old, compared to those of age matched WT controls. Furthermore, CC tissues of Akita mice at 12 and 24 wks had decreased nNOS expression levels compared to age matched WT mice. Consistent to these finding, our functional studies showed impairment of endothelium-dependent and nitrergic relaxation of 12 and 24 wk Akita vessels.

Systolic blood pressure was moderately elevated in 24 wk Akita mice, but not at earlier time points. Hypertension also has been reported in 24 wk old Akita mice accompanied with renal hypertrophy and dysfunction [52], but was not present in 16 wk old Akita mice, which also displayed renal dysfunction and elevated kidney Arg2 activity and expression [27]. Our present study showed elevated vascular arginase activity occurs weeks before there was elevation of blood pressure.

Regulation of vascular smooth muscle tone involves a delicate balance between relaxing and contractile factors. In our study, the sensitivity of aorta to PE-induced contractions was higher at both early and later stages of diabetes in Akita *vs* WT. This observation can be explained by reduced NO production from eNOS allowing greater contractile responses to PE. In contrast to aorta, increased contractile responses of CC to EFS, but not to PE, were observed in Akita mice at 12 and 24 wks compared to those of WT mice. Since the functional consequences of stimulation of sympathetic versus nitrergic nerves are quite different, we suggest that augmentation of contractile responses to EFS in CC of Akita mice are due to changes in prejunctional events, most likely to enhanced sympathetic nerve function.

In summary, the current study validates the Akita mouse as a key animal model of spontaneous diabetes and that offers insight into mechanisms involving impairment of vascular function in diabetes. The present study shows that over a time-course of 24 wks of diabetes in Akita mice, both aortic and cavernosal tissues exhibit increased arginase activity and expression (Arg1 in aorta, and Arg1 and Arg2 in CC), contributing to impairment of endothelial and nitrergic function and reduced NO production. Use of this genetic animal model that closely mimics human diabetes can significantly improve our understanding of mechanisms involved in diabetes-induced endothelial and nitrergic dysfunction and further facilitates therapeutic targets for normalizing vascular function in diabetes.

## Supporting Information

Figure S1 **Increased Arginase Activity in Liver and Kidney from Akita Mice.** Increased arginase activity was observed in liver (at 12 and 24 wks of age) and kidney (4, 12 and 24 wks of age) from Akita compared with those of age matched WT mice (panel **A** and **B**). Data represent the mean ± S.E.M. of 4–5 experiments. * *p*<0.05; *** *p*<0.001compared to its WT group.
(TIF)

Figure S2 **Endothelium-independent relaxation in aortas and in CC from age matched WT and Akita mice.** Concentration-response curves to SNP in aorta (panel **A**) and CC (panel **B**) tissues from age matched WT and Akita mice at 4, 12 and 24 weeks of age. Inset: pEC_50_ values. Maximal response (E_max;_ right panel) values derived from SNP-induced relaxation. Data were calculated as changes from the contraction induced by PE (1 and 10 µmol/l in aorta and CC, respectively) in each tissue, which was taken as 100%. Data represent the mean ± S.E.M. of 3–5 experiments.
(TIF)

Figure S3 **Sympathetic and nitrinergic nerve stimulation in aorta and corpus cavernosum (CC) from Akita mice.** Concentration-response curve to PE in aorta (0.001–10 µmol/l, panel **A–C**) and CC (0.001–100 µmol/l, panel **D–F**) from wild type (WT) and Akita mice. Sympathetic nerve stimulation induced by EFS (1–64 Hz) in CC of WT and Akita mice (panel **G–I**). Depletion of catecholamine stores by bretylium tosylate fully blocked contractile responses induced by EFS (panel **I**). Experimental values were calculated relative to the maximal changes from the contraction produced by KCl (80 mmol/l), which was taken as 100%. Data represent the mean ± S.E.M. of 4–5 experiments. * *p*<0.05 and ** *p*<0.01, compared to its concentration or frequency of WT mice.
(TIF)
