# Supplementary figures and images for: Akita Spontaneously Type 1 Diabetic Mice Exhibit Elevated Vascular Arginase and Impaired Vascular Endothelial and Nitrergic Function
Source: PLoS One. 2013 Aug 19;8(8):e72277. doi: 10.1371/journal.pone.0072277 (PMC3747112; doi:10.1371/journal.pone.0072277)

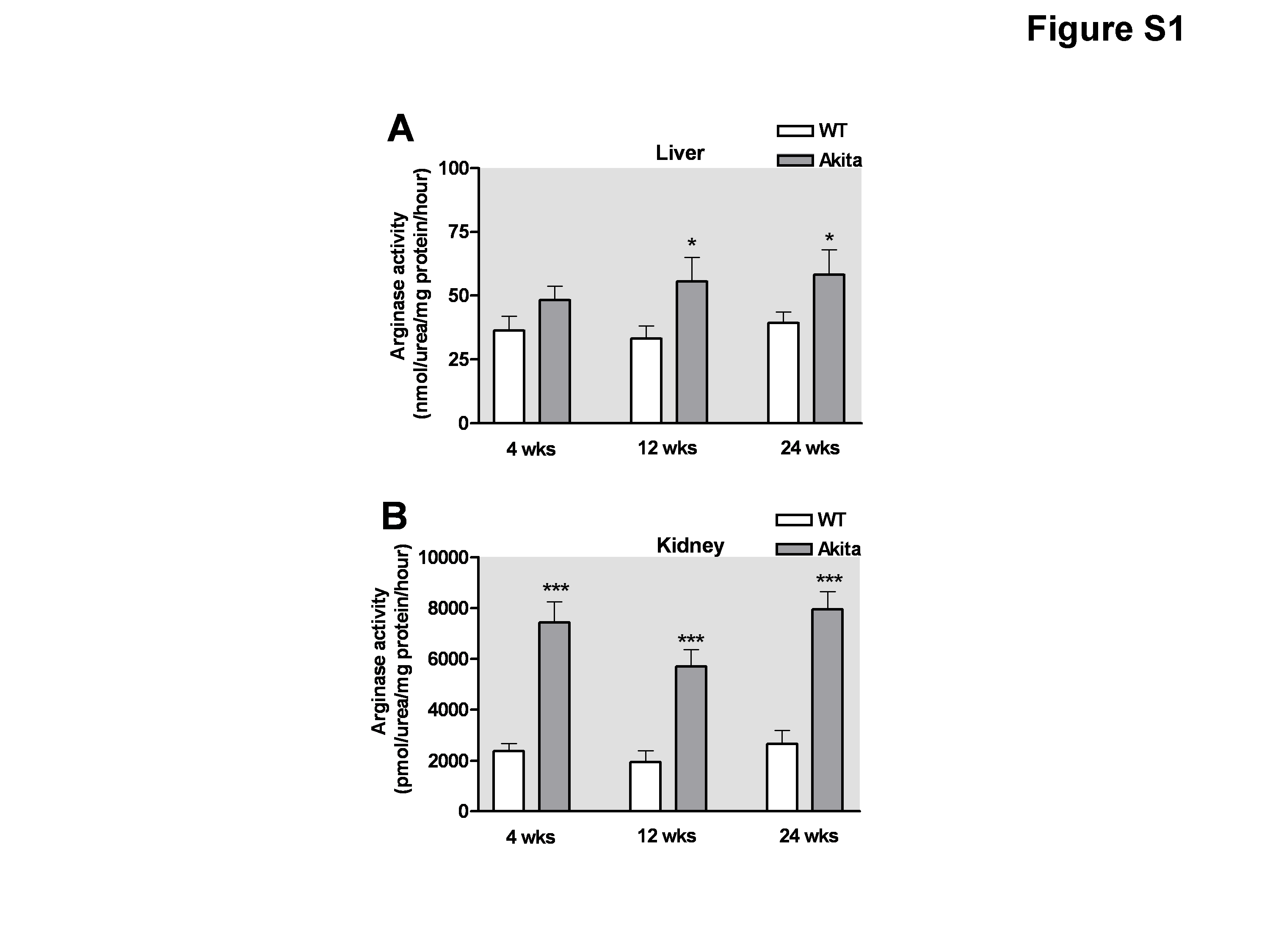

Supplement: Figure S1 — Increased Arginase Activity in Liver and Kidney from Akita Mice. Increased arginase activity was observed in liver (at 12 and 24 wks of age) and kidney (4, 12 and 24 wks of age) from Akita compared with those of age matched WT mice (panel A and B). Data represent the mean ± S.E.M. of 4–5 experiments. * p<0.05; *** p<0.001compared to its WT group. (TIF) [file pone.0072277.s001.tif]

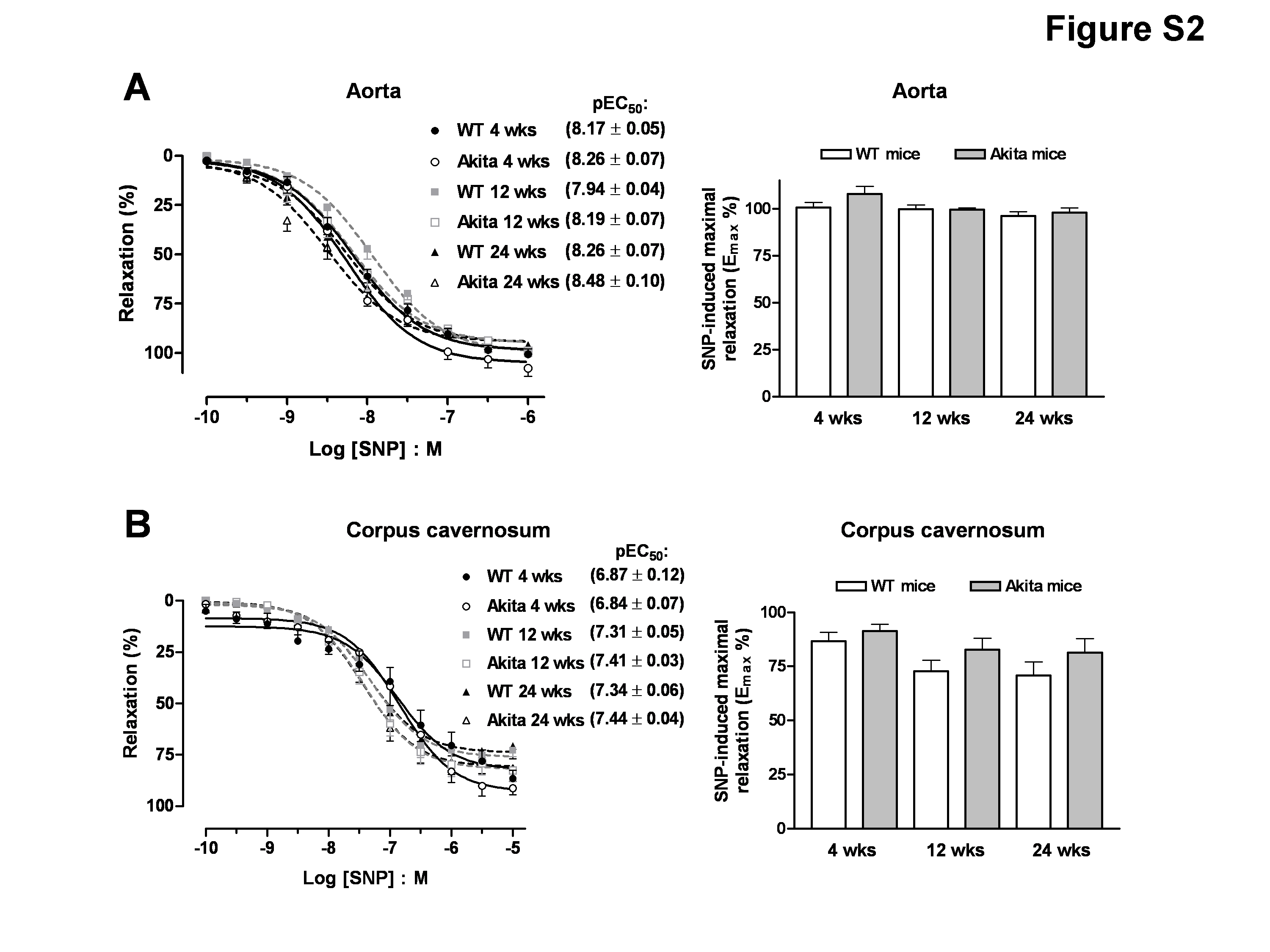

Supplement: Figure S2 — Endothelium-independent relaxation in aortas and in CC from age matched WT and Akita mice. Concentration-response curves to SNP in aorta (panel A) and CC (panel B) tissues from age matched WT and Akita mice at 4, 12 and 24 weeks of age. Inset: pEC50 values. Maximal response (Emax; right panel) values derived from SNP-induced relaxation. Data were calculated as changes from the contraction induced by PE (1 and 10 µmol/l in aorta and CC, respectively) in each tissue, which was taken as 100%. Data represent the mean ± S.E.M. of 3–5 experiments. (TIF) [file pone.0072277.s002.tif]

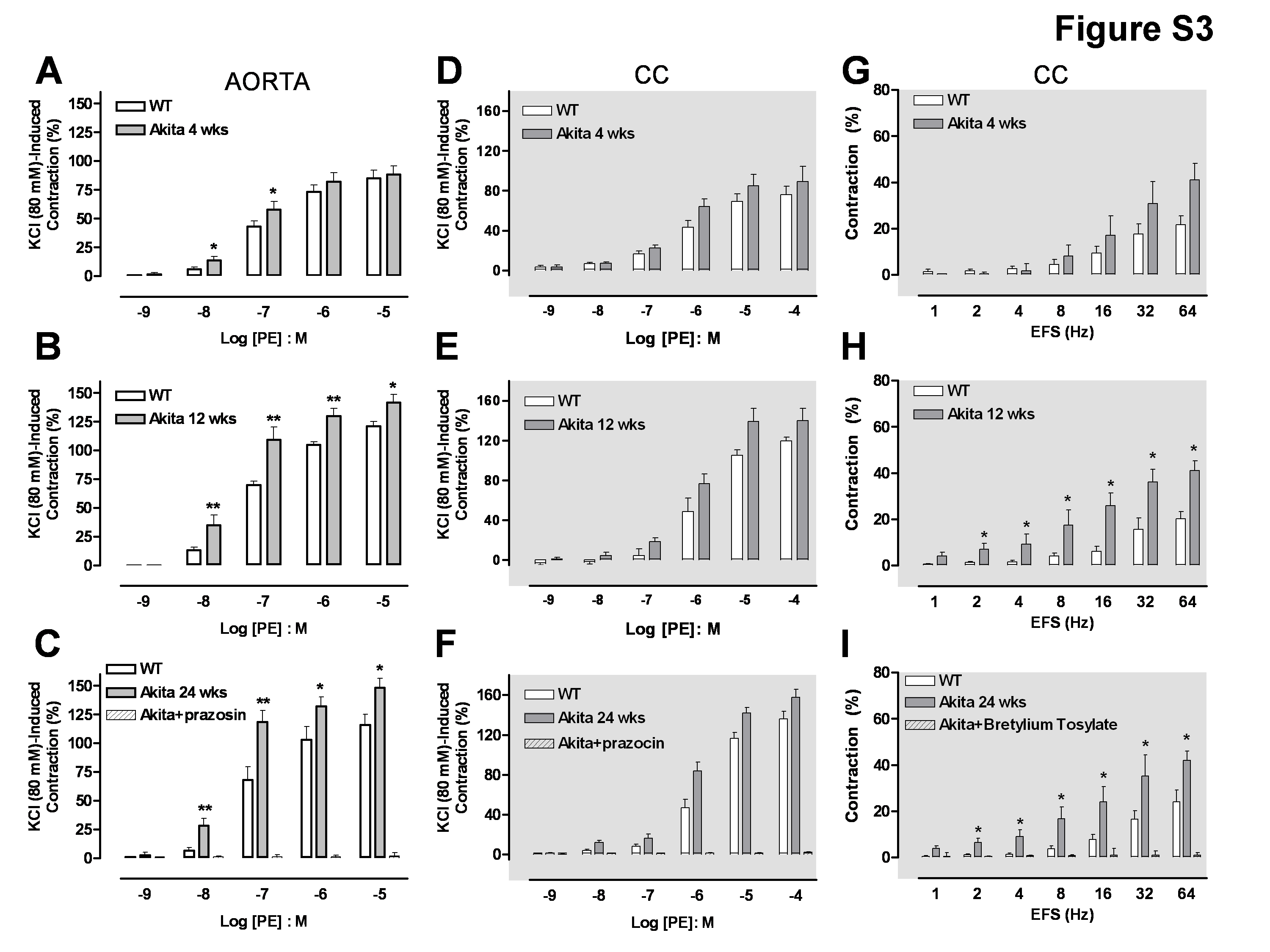

Supplement: Figure S3 — Sympathetic and nitrinergic nerve stimulation in aorta and corpus cavernosum (CC) from Akita mice. Concentration-response curve to PE in aorta (0.001–10 µmol/l, panel A–C) and CC (0.001–100 µmol/l, panel D–F) from wild type (WT) and Akita mice. Sympathetic nerve stimulation induced by EFS (1–64 Hz) in CC of WT and Akita mice (panel G–I). Depletion of catecholamine stores by bretylium tosylate fully blocked contractile responses induced by EFS (panel I). Experimental values were calculated relative to the maximal changes from the contraction produced by KCl (80 mmol/l), which was taken as 100%. Data represent the mean ± S.E.M. of 4–5 experiments. * p<0.05 and ** p<0.01, compared to its concentration or frequency of WT mice. (TIF) [file pone.0072277.s003.tif]
